# Audio-Visual Perception of 3D Cinematography: An fMRI Study Using Condition-Based and Computation-Based Analyses

**DOI:** 10.1371/journal.pone.0076003

**Published:** 2013-10-23

**Authors:** Akitoshi Ogawa, Cecile Bordier, Emiliano Macaluso

**Affiliations:** Neuroimaging Laboratory, Istituto di Ricovero e Cura a Carattere Scientifico, Santa Lucia Foundation, Rome, Italy; Harvard Medical School/Massachusetts General Hospital, United States of America

## Abstract

The use of naturalistic stimuli to probe sensory functions in the human brain is gaining increasing interest. Previous imaging studies examined brain activity associated with the processing of cinematographic material using both standard “condition-based” designs, as well as “computational” methods based on the extraction of time-varying features of the stimuli (e.g. motion). Here, we exploited both approaches to investigate the neural correlates of complex visual and auditory spatial signals in cinematography. In the first experiment, the participants watched a piece of a commercial movie presented in four blocked conditions: 3D vision with surround sounds (3D-Surround), 3D with monaural sound (3D-Mono), 2D-Surround, and 2D-Mono. In the second experiment, they watched two different segments of the movie both presented continuously in 3D-Surround. The blocked presentation served for standard condition-based analyses, while all datasets were submitted to computation-based analyses. The latter assessed where activity co-varied with visual disparity signals and the complexity of auditory multi-sources signals. The blocked analyses associated 3D viewing with the activation of the dorsal and lateral occipital cortex and superior parietal lobule, while the surround sounds activated the superior and middle temporal gyri (S/MTG). The computation-based analyses revealed the effects of absolute disparity in dorsal occipital and posterior parietal cortices and of disparity gradients in the posterior middle temporal gyrus plus the inferior frontal gyrus. The complexity of the surround sounds was associated with activity in specific sub-regions of S/MTG, even after accounting for changes of sound intensity. These results demonstrate that the processing of naturalistic audio-visual signals entails an extensive set of visual and auditory areas, and that computation-based analyses can track the contribution of complex spatial aspects characterizing such life-like stimuli.

## Introduction

Three-dimensional movies are becoming popular both for cinema projections as well as for home-based entertainment. Stereoscopic viewing together with multi-channels sound systems (e.g. 5.1ch surround) provide us with an enhanced perception of space and can augment the “sense of reality” during the movie watching [1]. Cinematographic stimuli have been previously employed to investigate brain activity associated with the processing of complex visual and auditory stimuli [2]–[6]. Functional imaging using naturalistic stimuli can help us to corroborate the findings of traditional laboratory paradigms that employ well-controlled but simple and stereotyped stimuli, thus extending the relevance of these to brain functioning in real life [7]. However, to our knowledge, previous neuroimaging studies have not yet used 3D vision with surround sounds (stereoscopy and multi-source sounds), possibly overlooking the impact of high-order spatial information during audio-visual perception in life-like environments.

Visual depth perception in a natural scene depends mainly on binocular disparity, which corresponds to the horizontal difference of the images that the two eyes receive [8]. A final goal of visual recognition with binocular disparity is to construct a unified 3D representation of the objects in the scene and their spatial relations. In the visual system, two computational processes play important roles for depth perception. One concerns the distance in depth between two locations in space, while the other relates to the surface geometry of 3D shapes [9]. The former, also referred as “absolute disparity”, is deduced from the interocular distance and vergence angles [10]. The latter defines the “disparity gradient”, which corresponds to the spatial offsets on the surface of 3D objects or at the boundary of objects at different depths.

Previous electrophysiology and neuroimaging studies associated the processing of stereoscopic stimuli with a widespread circuit of brain areas. A core region of this network is visual area V3A in the dorsal occipital cortex [11]–[13] that is thought to represent absolute disparity [14]–[16]. Other dorsal occipito-parietal areas, including the posterior parietal cortex (PPC) are also involved in the processing of stereoscopic signals [17]. In particular, PPC has been associated with the integration of disparity and monocular depth cues (e.g. texture and shading [17]) and with a generalized representation of 3D surface orientation [18]–[21]. Brain areas along the ventral occipito-temporal pathway have been also implicated in 3D processing. The inferior temporal (IT) cortex contains neurons selective for the direction of 3D curvature (vertical or horizontal) and the in-depth boundaries of objects [22], hence representing disparity gradients [23], [24]. A putative human homologue of the monkey IT cortex is the lateral occipital complex (LOC) that functional imaging studies traditionally associated with object processing [25]–[28]. A few previous fMRI studies in humans showed that objects defined by disparity gradients can activate the LOC [29]–[31].

Our understanding of brain areas involved in 3D processing comes primarily from studies that used very simple and stereotyped stimuli. For example, random dot stereograms (RDS) have been used to generate geometric 3D structure (e.g. planes, cones; see [13], [32], [33]). Albeit well-controlled, this technique does not allow presenting complex and naturalistic stimuli such as 3D photos or 3D movies. Moreover, natural 3D perception involves multiple cues that dynamically change and may interact with other aspects of the visual stimuli (e.g. motion [34], [35], or objects [36], [37]). Accordingly, the investigation of brain activity with complex 3D stimuli is important to confirm and extend results obtained with standard, well-controlled but unnaturalistic experimental paradigms.

Analogous limitations have characterized fMRI studies of auditory spatial processing that typically made use of simple and repeated sounds, such as pure tones or noise stimuli (e.g. [38]–[42]). Simple auditory stimuli presented thorough headphones permit subtle manipulations of specific parameters, e.g. the interaural level difference (ILD), the interaural time difference (ITD) and/or spectro-temporal characteristics. These, sometimes combined with head-related transfer functions in order to increase spatial perception (e.g. [43], [44]), revealed the role of posterior auditory areas in auditory spatial processing (e.g. the planum temporale, PT [45], see also [46]). Nonetheless, auditory presentation via headphones lacks the richness of spatial percept generated by complex sounds that originate from multiple locations [47], [48]. One notable research is the positron emission tomography study by Zatorre et al. [49], who used a circular array of speakers to present complex sounds from multiple positions (see also [50], which presented combinations of complex stimuli from a single external location). The results confirmed the role of the PT in auditory spatial progressing, but also emphasized the impact of presenting complex sounds from multiple locations by showing that PT does not respond to noise stimuli presented at a single location (cf. Exp 1 in [49]).

**Figure 1:**
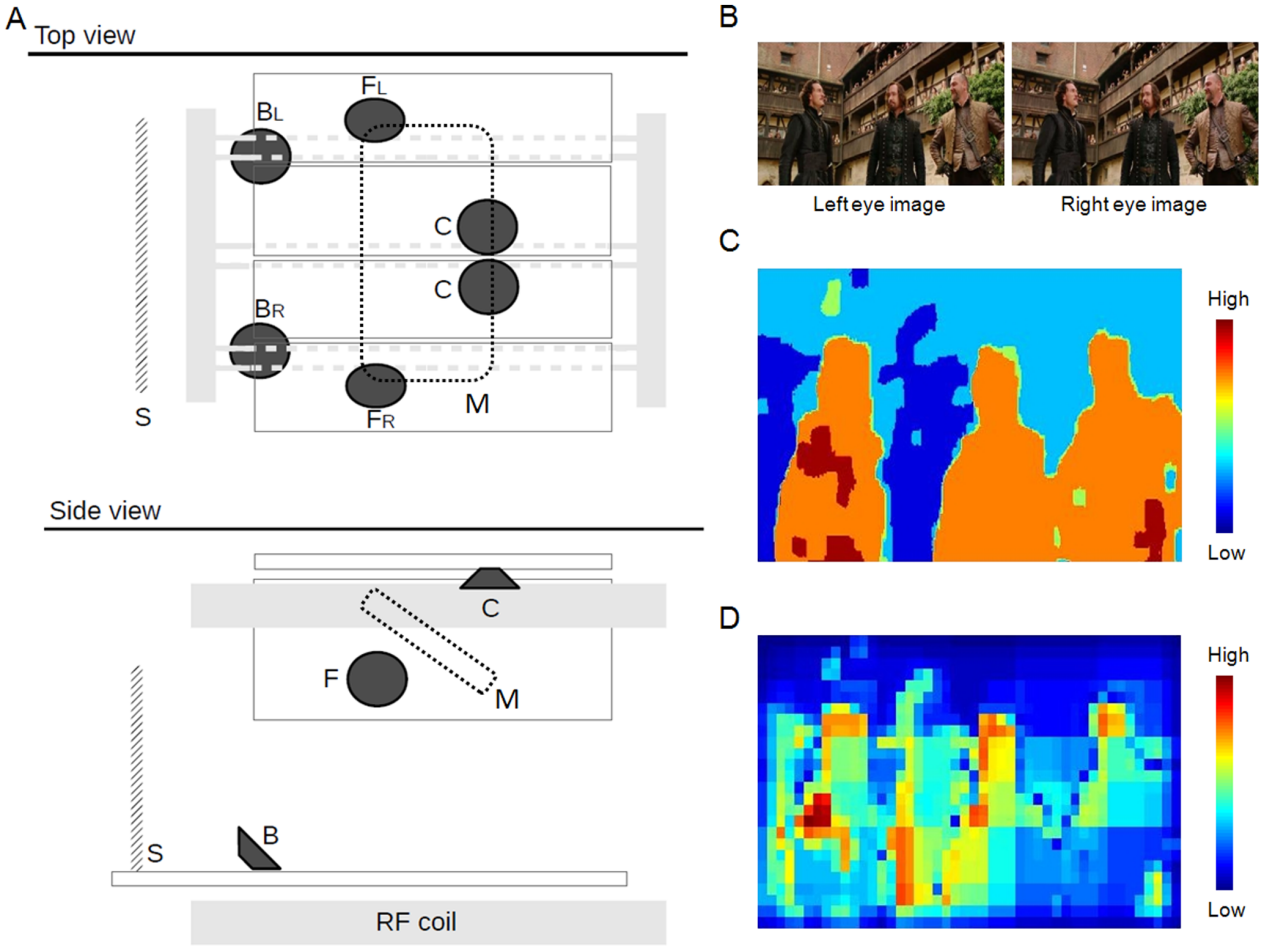
The multi-speakers system and the computation of absolute disparity and disparity gradient. **A**. Schematic illustration of the multi-speakers system used for sound-surround stimulation in the scanner. The system utilizes five independent sound-lines: a central channel (C: comprising two speakers delivering the same signal); two front channels (F_L_/F_R_) and two back channels (B_L_/B_R_). The drawings also show the approximate position of the screen (S) and of the mirror (M) used to show the visual stimuli. **B**. An example of a video frame, with the two different images for the left and right eye. The left and right images were projected thought a linear polarizer and were perceived as a single 3D image using a passive eyewear. **C**. The corresponding map of “absolute” disparity, computed using the algorithm of HL-SIFT flow [52]. **D**. The disparity “gradient” map associated with the same frame. This was computed by extracting the local intensity contrast of the absolute map, via Gaussian pyramid decomposition (see Methods section).

In the present study we made use of an apparatus that allowed us presenting complex sounds from multiple sources in the MR scanner (cf. Fig. 1A), concurrently showing visual stimuli in 3D. In the first experiment, we investigated the effect of multi-sources surround sounds and stereoscopic vision using a standard condition-based design that crossed factorially mono/surround sounds and 2D/3D vision. In the second experiment, we sought to track brain activity associated with time-varying aspects of the stereoscopic visual input and the multi-sources complex sounds. We made use of computational models to index changes of absolute disparity and disparity gradients on frame-by-frame basis, and dynamic changes of auditory complexity of the surround sound. For the auditory modality this allowed us also to assess in a more specific manner the effect of the surround stimuli, which in the “surround vs mono” categorical comparison of the first experiment entailed changes along several acoustic factors, including increased sound intensity (see also methods, below). These indexes were used for analyses of the fMRI time-series, with the aim of exploring any functional specialization associated with complex visual and auditory spatial signals, here using naturalistic stimuli for the first time.

## Materials and Methods

### Procedure

The study comprised two experiments, including the same participants who were presented with naturalistic audio-visual stimuli (movie segments). The first experiment manipulated the spatiality of the viewing/listening condition: 2D vs. 3D vision, monaural vs. surround sounds. The four resulting conditions were presented within a block-design that was analyzed using a standard condition-based approach. The second experiment was carried out in the same scanning session, just after Exp 1. Exp 2 comprised four fMRI runs, two with continuous 3D-Surround stimuli and two with 2D-Mono stimuli. Here we present only analyses of the two runs including 3D-Surround stimuli. These were analyzed using computationally derived regressors indexing specific visual (absolute disparity and disparity gradients) and auditory (source complexity and source intensity) time-varying aspects of the complex stimuli. It should be noted that it would not be possible to perform any corresponding analyses of the 2D-mono fMRI runs, because all indexes of interest (the two visual disparity indexes and the complexity auditory index) would be equal to zero at all time-points. Finally, we sought to confirm the results of the computation-based analyses by re-analyzing the data of Exp 1 now including the computationally derived regressors as well.

### Subjects

Sixteen Italian subjects (aged 21–39, mean = 27.3 years, 12 females and 4 males) with no history of neurological or psychiatric illness participated in this study. They had normal or corrected-to-normal visual acuity and reported no difficulty of hearing. All subjects reported no problem to see the stereoscopic 3D visual stimuli (see also pre-scanning test, below).

### Ethics Statement

The Ethical Committee of Santa Lucia Foundation approved this study. The subjects gave written informed consents prior to the scanning session.

### Stimuli

The audio-visual stimuli were created using the 3D Italian version of the movie *The Three Musketeers* (distributed by Constantin Film AG Frankfurt, Germany). For Exp 1, we used two segments of the movie, each with a duration of 10 min and 24 s. Each segment was divided into 16 blocks with a variable duration ranging between 26 and 57 s. The transitions between blocks always corresponded to a change of scene in the movie. We constructed four versions of each block: 3D-Surround, 3D-Mono, 2D-Surround, or 2D-Mono, corresponding to our conditions of interest. Each subject was presented with the same sequence of blocks, i.e. coherent with the movie storyline, but the viewing/listening conditions were fully counterbalanced across subjects using a balanced Latin square design. Accordingly, any differential activation as a function of the viewing/listening condition cannot be attributed to some un-controlled systematic difference between the different blocks.

For Exp 2, we used two different consecutive segments of the movie each lasting for 4 min 30 s. Both segments were presented twice during fMRI. The first segment was presented first in 3D-Sourround and then in 2D-mono, while the second segment was presented first in 2D-mono and then in 3D-Surround. This order was identical for all subjects and was chosen in order to counterbalance the effect of viewing/listening condition and the effect of the first/second presentation in the repetition. This specific protocol was chosen to compare 3D-Surround and 2D-Mono conditions using data-driven analysis methods, which will be reported elsewhere. Here we report computation-based analyses of the two 3D-Surround fMRI runs only (see also Procedure, above).

The video and the multi-channels sound track were extracted from a blue-ray disk using DVDFab (www.dvdfab.com), cropped and concatenated using ffmpeg (www.ffmpeg.org). The video was saved in MPEG-4 format at a rate of 24 Hz. The sound track was extracted twice, either with 6 channels (5.1 Surround) or with 1 channel (Mono). All sounds were saved in a Waveform Audio File format at a rate of 48000 Hz. The single mono-channel was presented over headphones during all experimental conditions (see below), while the 5 surround channels (the “subwoofer” channel was excluded) were delivered via external speakers in the surround conditions (see also Fig. 1A).

During fMRI scanning the presentation of the visual and the auditory stimuli was controlled using “psychophysics toolbox” [51] running on Matlab 7.1 (Mathworks, Inc.). The visual stimuli were presented using a LCD projector (NEC Corp., NP216G) operating at 120 Hz, which was synchronized with a linear polarizer (DepthQ®, Lightspeed Design Inc.). The visual stimuli were projected on a semi-opaque screen positioned inside the magnet, which the subjects viewed via a mirror system (see Fig. 1A). The subjects wore a MR-compatible passive 3D eyewear allowing them to view the polarized images just with the left or the right eye. This generated the stereoscopic stimuli, when different images were projected to the two eyes (i.e. in the 3D conditions). Presenting the same image to both eyes generated the 2D condition.

The auditory stimuli were presented via a multi-speakers system that was built in-house (Fig. 1A). This consisted of six MR compatible piezo-speakers (ISL Products International, Ltd., SPK-PZ94 HES, please see www.islproducts.com for detailed specification of the frequency response) that were used to deliver the center channel (two speakers “C” positioned centrally, behind the mirror, which emitted the same sounds), the two L/R front channels (speakers “F_L_/F_R_” positioned symmetrically at approximately 40° to the left and to the right of the mid-sagittal plane), and the two L/R back channels (speakers “B_L_/B_R_” positioned left and right at approximately 20° from the mid-sagittal plane, behind/above the head of the subject, see Fig. 1A). In addition, the system included a headphone-line that was used to deliver the mono signal to both ears (cf. also extraction of the auditory tracks, above). In the Mono condition only the headphone-line was active, while in the Surround condition the sounds were presented from all speakers plus the headphone. Thus, the Surround condition compared to the Mono condition entailed an overall increase of sound intensity, as well as a variation of the auditory input with respect of several other acoustic dimensions (please see section below about indexes of “auditory intensity”, and the Discussion section about other acoustic changes associated with the surround stimulation).

Furthermore, it should be considered that the scanner noise may have masked some frequencies of sounds presented from the external speakers, and possibly also from the headphone. In a separate testing session, we investigated this issue by recording the noise of the EPI sequence using a microphone placed inside the headphone (sampling rate 48 kHz). The power spectrum density of the EPI noise was found to peak at 984, 1000, and 1016 Hz. The frequency band of scanner noise was very narrow (±0.01 Hz). Accordingly, the EPI noise may have masked the experimental auditory signals (speakers+headphone) at around 1000 Hz.

Before starting Exp 1, we confirmed that the subject could see the entire visual image and that the sounds were audible against the noise of the echo-planar imaging sequence. The participants watched a one-minute segment of the movie both in 2D-Mono and 3D-Surround conditions. They were asked to confirm that they could see the 3D images and hear the surround sounds against the scanner noise in 3D-Surround condition. While we cannot not exclude the possibility of weak amblyopia in some of the subjects (e.g. strabismic amblyopia, which is difficult condition for stereoscopic viewing), this ensured that all the subjects could see the stereoscopic 3D visual input. Further, in the after scanning debriefing, we confirmed that all the subjects had seen the 3D images and heard the surround sounds.

#### Computational indexes of visual disparity

We extracted indexes of absolute disparity and disparity gradients, on a frame-by-frame basis (Figs. 1B–1D). First, each pair of images was submitted to the scale-invariant feature transform flow (SIFT flow [52]). We considered the horizontal layer (HL) SIFT flow map that was computed as the horizontal “optical flow” of SIFT descriptors (Fig. 1C) characterizing local features in images [53] in a coarse-to-fine matching scheme [52]. Under the assumption that – on average – subjects fixated on the zero disparity plane (ZDP), for each frame we defined the absolute disparity as the mean of the absolute values of the entire HL-SIFT flow map.

The HL-SIFT flow map was also used to index the disparity gradient. This was defined as the local contrast of within each HL-SIFT flow map (see Fig. 1D). The local contrast was computed by applying a Gaussian pyramid decomposition to the intensity values of the HL-SIFT flow map [54]. The method decomposes each map in 9 levels and computes intensity contrasts at different spatial scales, which are then combined into a single map (see also [55], which used this approach to index other visual features). For each frame, the disparity gradient was indexed averaging all the values of the corresponding contrast map.

Frame-by-frame values of absolute disparity and disparity gradient were re-sampled at the temporal resolution required for the regression analyses by averaging the disparity values over all frames of each MR volume (Repetition Time = 2.08 s). Finally, the re-sampled vectors were convolved with the SPM8 hemodynamic response function (HRF) in order to generate the final regressors.

#### Computational indexes of the complex surround sounds

For each of the 5 sound signals we computed an index of source complexity that quantifies the dissimilarity of the surround-signals with respect to the mono-signal delivered over the headphones. The complexity index was estimated using the inverse of the cosine similarity [56] (see also the equation below). The angular difference between two sounds waves can range between 0°, when the sounds are identical, and 180° when the two signals have the same shape but opposite directions. An angle of 90° indicates orthogonality between the two signals, implying a rich sound environment and greater sound spatiality (cf. [49]). For each sound signal (*S_i_*, with *i* = 1…5) we computed the complexity index with respect of the headphone signal (*S_0_*), using time–windows of one TR (2.08 s):
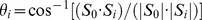
where, dots indicate inner product and straight brackets indicate absolute value.

The complexity index is unaffected by the signal amplitude, i.e. the sound intensity. Because the speaker sounds were added to the headphone sound during surround stimulation, the surround conditions entailed an increase of sound intensity compared with the mono conditions in Exp 1, and generated intensity-related variability over time in Exp 2. Therefore, we introduced an additional index of “auditory intensity” in order to map intensity-related changes of brain activity, and – most importantly – to identify brain regions responding to “auditory complexity” after having accounted for any variance associated with the changes of sound intensity. An “intensity index” was computed for each channel (5 surround channels+headphone). Specifically, we considered the sound intensity contrast extracted using the same multi-scale approach adopted for the visual modality (see also [55]). Each 2.08 s sound segment was first submitted to Fast Fourier analysis [57]. The resulting spectrogram was analyzed with the Gaussian pyramids to extract the intensity contrast at different scales. These were then combined into a single map. The intensity index (one value for each TR) was defined as the mean of all values in the contrast map. All the 11 auditory-related indexes (5 complexities and 6 intensities) were convolved with the HRF to generate the final regressors for the fMRI analyses.

These “auditory intensity” indexes reflect auditory contrasts in the time-frequency domain that we previously found to co-vary with activity in the auditory cortex [55]. For completeness, we also performed additional analyses using a simpler measure of sound intensity: i.e. the route mean square (RMS), which reflects the power of the sound only in the time domain. Again, we computed an RMS-intensity index for each channel and, after convolution with the HRF, used these as intensity-related regressors for the fMRI analyses.

### Image acquisition

A Siemens Allegra (Siemens Medical Systems, Erlangen, Germany) 3T scanner equipped for echo-planar imaging (EPI) was used to acquire the functional resonance images. A head-sized quadrature volume coil was used for radio frequency transmission and reception. Mild cushioning minimized head movement. Thirty-two slices volumes were acquired using blood oxygenation level dependent contrast (192 mm×192 mm×120 mm, in-plane resolution = 64×64, pixel size = 3 mm×3 mm, thickness = 2.5 mm, 50% distance factor, TR = 2.08 s, TE = 30 ms), covering the entire cerebrum. We acquired 308 volumes for each fMRI run of Exp 1 and 134 volumes for each run of Exp 2. The first four scans of each run were discarded to ensure magnetization equilibrium.

### FMRI data analyses

We used SPM8 (Wellcome Department of Cognitive Neurology, University College London) to pre-process and analyze the imaging data. Standard pre-processing steps included slice-timing correction, realignment, normalization to the EPI template (voxel-size re-sampled to 3×3×3 mm^3^) and spatial smoothing using a Gaussian filter (FWHM = 8 mm).

We performed three sets of analyses: A) Standard condition-based analyses of Exp 1; B) Computation-based analyses of Exp 2, using the time-varying indexes associated with 3D-vision and Surround-sound; C) Computation-based analyses of Exp 1, seeking to confirm the results obtained in Exp 2.

#### Condition-based analysis of Exp 1

In Exp 1, the audio-visual stimuli were presented in four viewing/listening conditions: 3D-Surround, 3D-Mono, 2D-Surround, and 2D-Mono. For each subject, the general linear model (GLM) was used to fit the fMRI time series. The model included 4 conditions (variable duration blocks = 26–57 s) and the realignment parameters as effects of no interest. High-pass filters (512 s) were used to remove low frequency noise and data were pre-whitened by means of autoregressive model AR(1).

Random effects analysis at the group-level was carried out using a repeated-measures ANOVA that modeled the 4 conditions of interest and the main effect of subjects. Linear contrasts assessed the main effect of visual disparity (3D vs. 2D conditions), the main effect of surround sounds (Surround vs. Mono) and the interaction between the two factors. Statistical thresholds were set to cluster-level p-FWE = 0.05, corrected for multiple comparisons considering the whole brain as the volume of interest. The cluster size was estimated using a voxel-level threshold of p-unc. = 0.001. In addition, we specifically considered visual area V3A and superior parietal lobule (SPL) that previous studies associated with stereoscopy [11], [12], [58]. We assessed our contrasts within these areas using a small volume correction procedure (SVC [59]). The volumes of interest included 15-mm radius spheres centered at x = ±20, y = −90, z = 23 for V3A (i.e. the peak coordinates reported in [60]); and at x = 24, y = −64, z = 58 and x = −22, y = −62, z = 56 for SPL (see [58] and discussion section).

#### Computation-based analyses (Exp1 and Exp 2)

For each subject, the general linear model (GLM) included the two regressors coding for visual disparity (absolute and gradient) and 11 regressors coding for auditory complexity and intensity. For Exp 1, the GLM included also the block-effects corresponding to the four different stimulation conditions (cf. condition-based analyses, above). Moreover, the visual regressors were adjusted so that each regressor was equal to zero during 2D stimulation and the total sum of each regressor was also zero. In essence, we orthogonalized the visual disparity indexes with respect of the block effect of 3D stimuli. Accordingly, the computation-based analysis of Exp 1 assessed the effect of the time-varying disparity indexes over and above any sustained/blocked effect of the 3D stimulation. The same procedure was applied to the auditory indexes, which therefore tested for the effects of auditory complexity and intensity having accounted for any sustained/blocked effect of the surround stimulation. For both experiments, high-pass filters (cut-off = 512 s) were used to remove low frequency noise, and linear contrasts were used to average the parameter estimates associated with each of the 13 regressors across the two fMRI runs.

For both datasets (Exp 1 and Exp 2), we used two separate one-sample t-tests to assess the significance of the visual indexes (absolute disparity and disparity gradient) at the group level. Statistical thresholds were set to cluster-level p-FWE = 0.05, corrected for multiple comparisons considering the whole brain as the volume of interest. The cluster size was estimated using a voxel-level threshold of p-unc. = 0.001.

The effect of the time-varying auditory indexes was assessed using two separate ANOVAs: one including the 5 parameter estimates corresponding to the complexity indexes, and the other with the 6 parameter estimates associated with intensity indexes. Because of the high correlation between the 5 complexity-regressors and between the 6 intensity-regressors, within each ANOVA we used F-contrasts testing for the combined effect of all complexity or intensity regressors. Initially, the F-contrasts were assessed at the same threshold of all the t- contrasts (p-FWE = 0.05, with cluster-level correction, cf. above). However, this revealed extremely large clusters of activation. Because statistical inference based on cluster-level correction entails an uncertainty about the localization of the effects within the significant clusters, we considered these initial results not sufficiently informative. Therefore, the final thresholds for the F-contrasts were set to p-FWE = 0.05, whole-brain corrected for multiple comparisons at the voxel-level (minimum cluster size = 20 voxels).

## Results

### Condition-based analysis (Exp 1 only)

**Figure 2:**
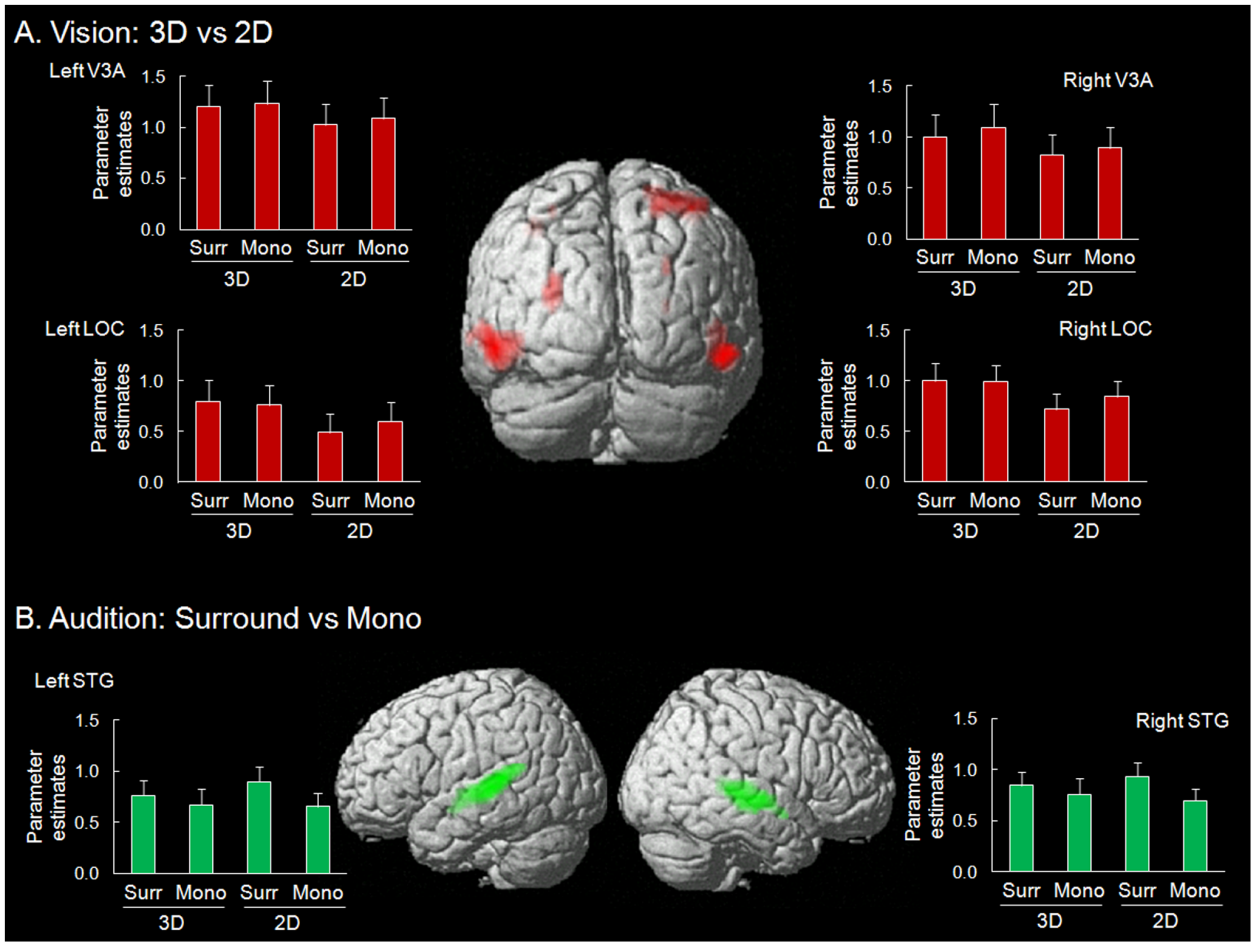
The results of condition-based analyses of Exp 1 (see **Table 1**), rendered on the brain template of SPM. **A**. Activation for the main effect of “3D>2D” visual stimulation. The signal plots show the parameter estimates in V3A and LOC, separately for the 4 experimental conditions. **B**. Activation for the main effect of “Surround>Mono” auditory stimulation. The signal plots show the parameter estimates in the superior temporal gyrus (STG). All activations are displayed at a threshold of p-unc. = 0.001. The signal plots show the average activity within each cluster, extracted using MarsBaR [81]. Parameter estimates are in arbitrary units, error bars are standard errors.

In Exp 1, we tested for brain activation associated with 3D viewing comparing blocks of 3D vs. 2D visual stimulation. This showed significant activation in left inferior temporal gyrus extending posteriorly to lateral occipital cortex (ITG/LOC) and a statistical trend in right ITG/LOC (p-FWE = 0.050 at cluster-level). Additional tests that specifically targeted area V3A showed a significant effect of 3D in both hemispheres (Table 1). Fig. 2A shows the signal plots in these areas, where the activity increased in the 3D conditions irrespective of Surround/Mono sounds. Although the parameter estimates associated with the mono conditions were numerically larger than in the surround condition, the activity in the mono conditions was not significantly different than the activity in the surround conditions neither in 3D nor in 2D viewing condition. Using small volume correction, the condition-based analysis also showed an effect of 3D in right anterior SPL (aSPL, Fig. 2A and Table 1), where subsequent computation-based analyses revealed an influence of absolute disparity (cf. below).

**Table 1:** Summary of brain activation in the condition-based analysis in Exp 1.

Contrast Regions	MNI coordinates of peak			z-score (peak)	p-value (cluster)	Number of voxels
	*x*	*y*	*z*			
3D>2D						
L ITG/LOC	−51	−60	−7	4.05	0.019	168
R ITG/LOC	54	−51	−10	4.06	0.050	124
L V3A*	−27	−81	17	3.77	0.008	46
R V3A*	27	−78	29	3.23	0.047	9
R aSPL*	33	−60	62	3.85	0.004	100
Surr>Mono						
L STG/STS	−57	−30	5	4.78	<0.001	461
R STG/STS	60	−18	−4	4.40	<0.001	371

* p<0.05, small volume FWE corrected at voxel-level. L = left, R = right.

The main effect of surround sound (Surround vs. Mono) revealed activation of the superior temporal gyrus (STG, see Fig. 2B and Table 1), extending into both the superior and the inferior banks of the superior temporal sulcus (STS). In both hemispheres, the activation clusters comprised the Heschl's gyrus in the superior/dorsal part of the STG (with activation of TE sub-regions TE1.0, TE1.1 and TE1.2) and included more posterior regions in the planum temporal (PT). The reverse contrast (Mono>Surround) showed no significant activation, even at a lower, uncorrected threshold of p = 0.005. We also tested for the interaction between stereoscopic vision and surround audition, but this did not reveal any significant effect.

### Computation-based analyses of visual disparity

**Figure 3:**
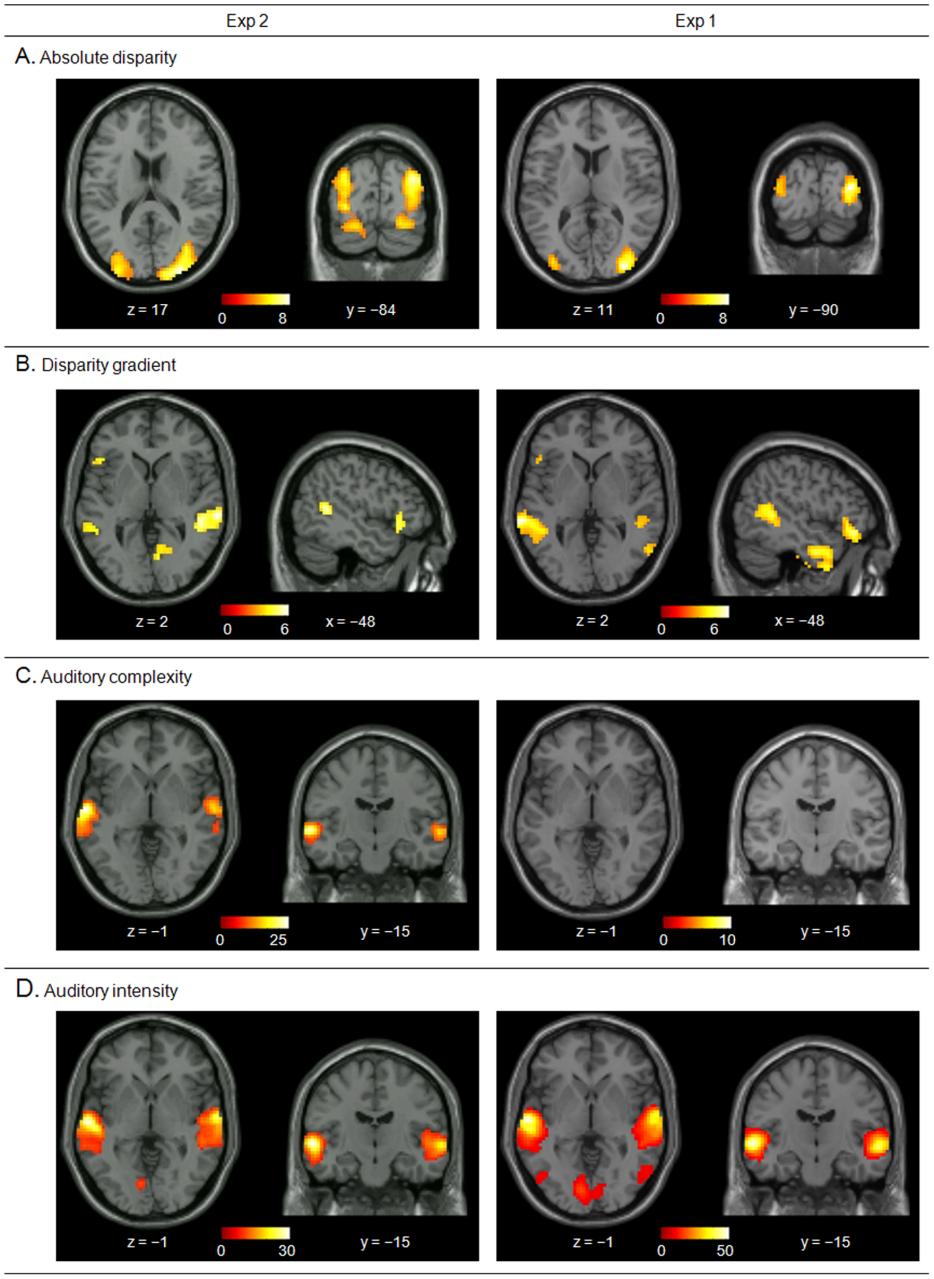
The result of computation-based analysis in Exp 2 and Exp 1 (see **Table 3**), shown on transverse and coronal sections of the SPM template, in neurological convention. **A**. Activations associated with the absolute disparity index, with significant effects in V3A (Exp 2 and Exp 1), plus the pSPL and the fusiform gyrus in Exp 2 only. Activations are displayed at a threshold of p-unc. = 0.001; colorbars show t-values. **B**. Activations associated with the disparity gradient index, showing consistent effects in the posterior middle temporal gyrus bilaterally (pMTG) and the left inferior frontal gyrus (IFG) both in Exp 2 and Exp 1. Activations are displayed at a threshold of p-unc. = 0.001; colorbars show t-values. **C**. Activations associated with the auditory “complexity” index that, in Exp 2, included the planum temporale (PT) and TE sub-regions in the auditory cortex (cf. Table 4; and see Fig. 4, for a detailed view). These effects in auditory cortex did not replicate in Exp 1, after accounting for the block effect of “surround vs. mono” presentation (cf. sections on the right). Activations are displayed at a threshold of p-FWE-corr. = 0.05; colorbars show F-values. **D**. Activations associated with the auditory “intensity” index, revealing effects in STG and STS, plus some influence also in the occipital visual cortex (cf. results). Activations are displayed at a threshold of p-FWE-corr. = 0.05; colorbars show F-values.

We examined the visual effects of absolute disparity and disparity gradient, first assessing these using the continuous 3D-Surround data of Exp 2, and then seeking to confirm our results in Exp 1. In Exp 2 we found a positive co-variation between absolute disparity and the BOLD signal in V3A (Fig. 3A, left panel), with activation extending dorsally to the posterior SPL (pSPL) and ventrally in the fusiform gyrus (see Table 2). The parietal clusters were more medial and dorsal compared to the blocked effect of 3D vs. 2D in Exp 1 (cf. peaks in Table 1 vs. Table 2). However, using the same region of interest as in Exp1 (aSPL-ROI) revealed significant effects of absolute disparity in both hemispheres (p-FWE<0.05), thus implicating the same SPL region [58] in the blocked-effect of 3D stimuli and the time-varying effects of absolute disparity. By contrast, the disparity gradient index revealed co-variation with activity in the posterior MTG (pMTG) and in the left inferior frontal gyrus (IFG, see Fig. 3B and Table 2), neither of which activated in the standard condition-based analysis. We did not find any significant effect of vision in auditory areas, neither in Exp 1 (block- and computation-based analyses) nor in Exp 2 (computation-based analyses) (cf. below).

**Table 2:** Summary of brain activation in the visual computation-based analyses.

Feature Regions	Exp 2						Exp 1					
	Peak in MNI			*z*-score (peak)	*p*-value (cluster)	Number of voxels	Peak in MNI			*z*-score (peak)	*p*-value (cluster)	Number of voxels
	*x*	*y*	*z*				*x*	*y*	*z*			
Absolute disparity												
L V3A	−30	−96	14	4.53	<0.001	1126	−36	−87	11	3.94	0.045	73
L FG	−24	−45	−16	5.14								
L pSPL	−9	−66	65	3.67								
R V3A	30	−93	17	5.15	<0.001	1162	33	−90	11	5.11	<0.001	334
R FG	30	−42	−13	5.12								
R pSPL	9	−60	68	4.41	0.040	67						
Disparity gradient												
L pMTG	−48	−48	8	4.31	0.023	88	−69	−36	2	4.79	<0.001	856
L IFG	−48	24	−4	3.79	0.017	95	−48	33	−10	4.32	0.011	91
R pMTG	66	−33	2	4.38	<0.001	249	63	−57	14	4.13	0.027	74
							48	−33	−4	4.13	0.006	103
R CG	9	−66	7	4.37	<0.001	314						
R TP							51	9	−28	4.33	0.046	64
R ITG							42	3	−49	3.85	0.022	78

FG = Fusiform gyrus, CG = Calcarine Gyrus, TP = Temporal pole.

The dissociation between absolute disparity and disparity gradient was largely confirmed applying the computation-based analysis to the data of Exp 1: activity in bilateral V3A co-varied with the absolute disparity index, while the disparity gradient was associated with the bilateral pMTG and the left IFG (see Figs. 3A and 3B and Table 2, columns on the right). It should be noted that this analysis included both the time-varying disparity indexes as well as the blocked-effect of 3D vs. 2D condition (cf. methods). This may explain the lack of any disparity effect in the SPL, where sustained components may have contributed to the effects observed in Exp 2 (see also discussion section).

### Computation-based analyses of auditory complexity

Next, we turned to the assessment of the auditory complexity and intensity indexes. In Exp 2, the F-contrast testing for the overall effect of the 5 complexity indexes revealed significant effects in the STG and STS bilaterally (Fig. 3C, left panel, and Table 3), plus a cluster in cuneus. In the STG, the effect of complexity comprised the lateral part of the Heschl's gyrus and extended posteriorly to the PT. The intensity index showed a similar pattern of activation (see Fig. 3D), but now also including the medial part of Heschl's gyrus (HG).

**Table 3:** Summary of brain activation in the auditory computation-based analyses.

Feature Regions	Exp 2						Exp 1					
	Peak in MNI			*z*-score (peak)	*p*-value (cluster)	Number of voxels	Peak in MNI			*z*-score (peak)	*p*-value (cluster)	Number of voxels
	*x*	*y*	*z*				*x*	*y*	*z*			
Complexity												
L STG/STS	−63	−15	−1	>8	<0.001	397						
R STG/STS	63	−9	−4	6.80	<0.001	190						
Cuneus	0	−93	17	6.42	<0.001	142	3	−90	17	5.20	0.005	43
Intensity												
L STG/STS	−60	−15	−1	>8	<0.001	872	−60	−12	−4	>8	<0.001	1478
L MOG							−45	−75	8	7.33	<0.001	
R STG/STS	66	−12	−4	>8	<0.001	752	60	−12	−7	>8	<0.001	853
							51	−63	8	6.77	<0.001	233
L LG	−9	−75	−4	6.29	<0.001	49	−12	−81	−4	7.59	<0.001	1008
L SPL	−15	−75	47	5.59	0.001	94						

MOG = middle occipital gyrus, LG = lingual gyrus.

**Figure 4:**
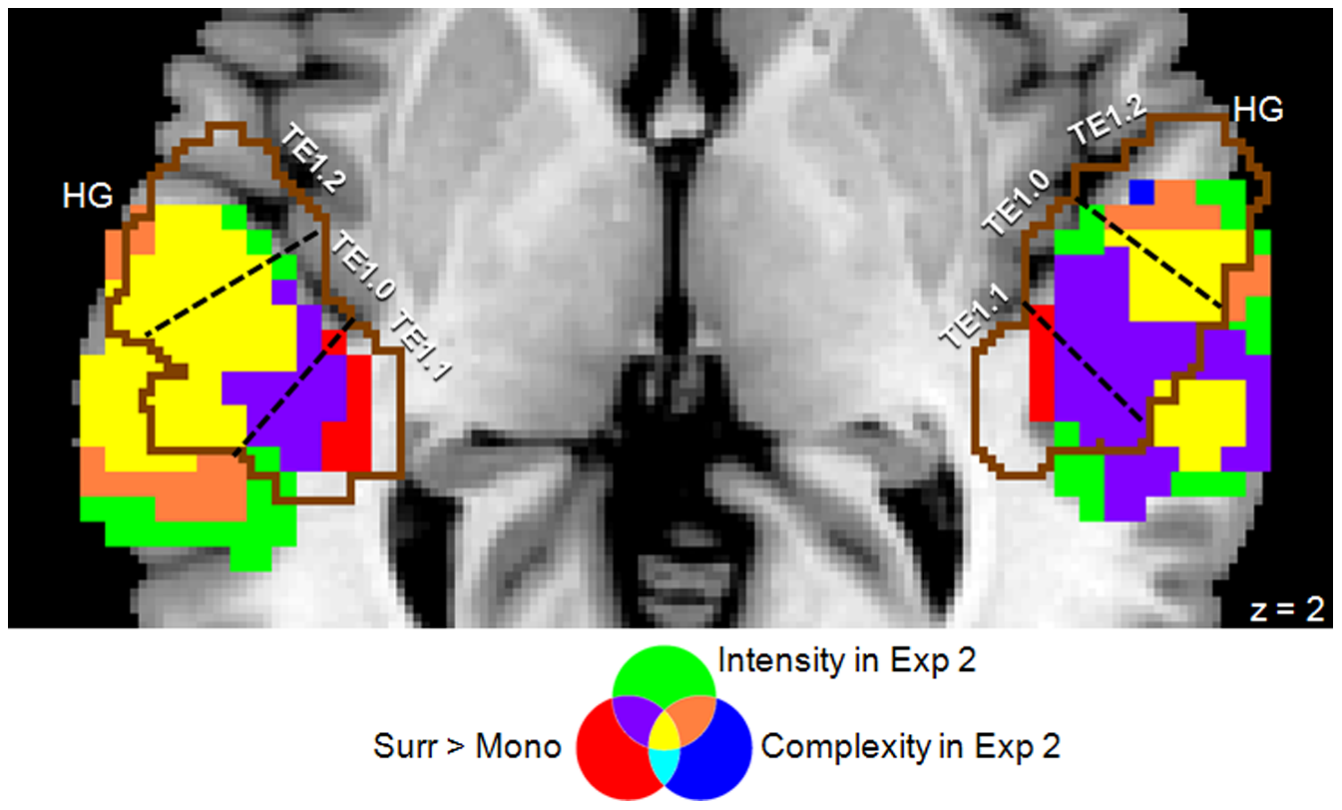
Detailed view of Heschl's gyrus (HG), with the layout of the different auditory effects in the auditory cortex (AC) and the planum temporale (PT). This shows that the blocked effect of surround sounds (in Exp 1) and time-varying effects of auditory intensity (in Exp 2) activated all TE sub-regions of AC plus the PT (in yellow and in violet). By contrast, the effect of auditory “complexity” activated the PT and only the most lateral part of AC (in yellow and in orange), without affecting area TE1.1. The broken lines show the borders between the TE sub-regions (TE1.0, TE1.1, and TE1.2) estimated using the SPM anatomy toolbox [82]. Activations are displayed on axial section (z = 2).

Figure 4 shows the spatial distribution of the effects associated with complexity and intensity in the STG, in relation to the main effect of “Surround vs. Mono” (Exp 1) and the TE sub-regions. The effect of intensity fully overlapped with the blocked-effect of surround sound and included all three TE sub-regions (cf. also Table 4), plus the PT posterior to the HG. By contrast, the effect of complexity activated only the most lateral part of HG (mainly area TE1.2, see also Table 4) and, again, the PT posterior to the HG.

**Table 4:** The classification of auditory activation related to the surround sound observed in the condition-based analysis and computation-based analyses, using the SPM anatomy toolbox [82].

	Left			Right		
Condition, Exp 1						
Surr>Mono	TE1.0	TE1.1	TE1.2	TE1.0	TE1.1	TE1.2
	29.1%	52.0%	21.7%	29.0%	33.4%	4.9%
Computation, Exp 2						
Complexity	TE1.0	TE1.1	TE1.2	TE1.0	TE1.1	TE1.2
	15.5%	0.1%	37.2%	10.1%	0%	40.8%
Intensity	TE1.0	TE1.1	TE1.2	TE1.0	TE1.1	TE1.2
	49.0%	39.3%	59.4%	75.9%	13.8%	44.8%
Computation, Exp 1						
Complexity	TE1.0	TE1.1	TE1.2	TE1.0	TE1.1	TE1.2
	0%	0%	0%	0%	0%	0%
Intensity	TE1.0	TE1.1	TE1.2	TE1.0	TE1.1	TE1.2
	97.0%	99.0%	85.2%	72.9%	45.7%	78.9%

In Exp 2, the effect of complexity was found even after accounting for the time-varying changes of auditory intensity (see methods) indicating that in lateral auditory cortex and PT the sound complexity played a role over and above any effect of intensity. Nonetheless, the complexity index failed to reveal any significant activation of STG/STS in Exp 1 (see Fig. 3C, right panel). This suggests the presence of a sustained component that was removed when testing the effect of sound complexity in Exp 1 (cf. also absolute disparity in the SPL, above). On the other hand, the effect of intensity in STG/STS was also found in Exp 1 (see Fig. 3C, right panel) highlighting transient intensity-related responses over any sustained component.

For completeness, we also performed additional analyses using RMS-intensity as a time-varying index of sound intensity, rather than our main index based on contrasts in the frequency-time domain (cf. Methods). These revealed intensity-related effects comparable with our main results. In experiment 1, where the model included both the blocked/sustained-effects and the computational indexes, the peak F-values associated with the effect of RMS-intensity in auditory cortex were larger than those obtained using contrasts in the frequency-time domain: left STG/STS 72.33 (at *x* = −60, *y* = −12, *z* = −4) vs. 52.92; and right STG/STS 70.15 (at *x* = 63, *y* = −15, *z* = −7) vs. 44.40. On the other hand, in experiment 2 that included a continuous variation of the surround sounds, the additional RMS-intensity analyses shower weaker statistical effects than the spectrogram-contrasts: left STG/STS 12.59 (at *x* = −48, *y* = −24, *z* = −1) vs. 30.71; and right STG/STS 12.00 (at *x* = 63, *y* = −9, *z* = −4) vs. 25.52.

Finally, we should point out that, unlike the T-contrasts used for visual disparity, all auditory F-contrasts cannot distinguish between positive and negative co-variation between the auditory indexes and changes of brain activity. Because the sound signals delivered from the 5 external sources and the headphone were correlated (but note: not fully correlated, as this was used as a measure of “sound complexity”), the regressors associated with each of the 6 intensity indexes and each of the 5 complexity indexes were also correlated. As a consequence, the positive/negative parameter estimates of the ANOVAs cannot be interpreted unambiguously (see also [55], for a detailed discussion of this). Thus, we suggest that the finding of a significant influence of both auditory complexity and intensity within the visual cortex (cf. Figs. 3C and 3D) may be related to a reduction of activity in these areas when the sound intensity and complexity increased, but we also acknowledge that we cannot assess this directly because our models included correlated predictors/regressors.

## Discussion

We investigated the neural correlates of the audio-visual processing of 3D-surround cinematography using condition-based and computation-based analyses. The condition-based results showed activation of ITG/LOC, V3A, and right SPL during 3D viewing; and activation of auditory areas in Heschl's gyrus (HG) plus the planum Temporale (PT) for surround sounds. The computation-based analyses revealed that V3A and SPL were primarily involved in the processing of absolute disparity, whereas pMTG and left IFG were associated with local disparity gradients. Analyses based on computationally-derived auditory indexes showed that activity in the planum temporale, plus areas TE1.0 and TE1.2 co-varied with both sound complexity and intensity, while the signal in area TE1.1 co-varied with sound intensity only. Our results demonstrate that computation-based analyses can track complex spatial aspects of visual and auditory naturalistic stimuli. We confirmed traditional findings about the role of extra-striate areas for visual disparity and posterior auditory areas for auditory spatial signals; but also found dissociations between absolute and gradient disparity in dorsal vs. lateral posterior regions, and highlighted disparity-related effects in the inferior frontal gyrus.

The condition-based analysis of Exp 1 associated processing of binocular disparity with activation of LOC, V3A, and the right SPL. This is largely consistent with previous neuroimaging studies comparing simple visual stimuli with vs. without disparity ([11], [12], [14], [61], [62] for effects of disparity in lateral occipital regions). Analyses based on computationally-derived indexes of absolute and gradient disparity provided us with additional elements about the processing of disparity cues in naturalistic visual environments. These indexes were computed with the aim of characterizing two distinct aspects of depth processing: the “absolute” disparity index relates to the in-depth distance between the objects in the scene and the zero-disparity-point (ZDP); while the disparity “gradient” should primarily capture effects related to the 3D geometry of objects [17], [33], [58].

Activity in the dorsal occipital cortex (area V3A) and the SPL was found to co-vary with the absolute disparity index. The effect in V3A was observed both in Exp 2 and in Exp 1, with the latter accounting for (i.e. removing) any sustained effect of 3D vs. 2D. This suggests that depth-related responses in V3A reflected both sustained/blocked effects during the 3D presentation as well as transient changes reflecting the amount of visual signals originating away from ZDP. This may entail both changes over time/frames of the number of objects presented away from the ZDP, as well as variations of the distance of the objects from the ZDP. Both these effects would be consistent with previous data showing activation of V3A both for single planes away from the ZDP and for multiple planes/objects at different distances from the ZDP [11], [13]. In the dorsal occipital cortex, several other areas have been previously associated with disparity processing and depth perception. Here we report disparity effects in areas V3A, defined according to a priori volume of interest [60]. Nonetheless, we do not exclude that the clusters of activation – and in particular the effect of absolute disparity (see Fig. 3A) – may comprise also parts of other dorsal visual areas, such as V3B/KO or V7.

It should be noted that our study made use of cinematographic stimuli that contained both binocular and monocular depth cues (e.g. motion parallax, perspective, shade, texture, elevation). Previous studies eliminated the influence of monocular cues, for example, by presenting Random Dots Stereograms [11], [12], [33] that can be used to generate well-controlled depth structures such as flat planes or curvatures. Nonetheless, here, in Exp 1 the subtraction of 3D vs. 2D conditions should cancel out many of these monocular effects (common to both conditions), thus identifying brain activity associated with the processing of binocular cues and/or the integration of binocular and monocular cues (e.g. see [17]). Together with V3A, this subtraction revealed activation in a relatively anterior/lateral part of the superior parietal lobule (aSPL). The effect was found using an a priori volume of interest derived from the co-ordinates reported in [58], who compared 3D shape vs. 3D position. In that study, this anterior region did not activate when the stimuli were presented away from the ZDP but without any 3D shape (i.e. “3D position” condition vs. no disparity), which instead activated more posterior regions in parietal cortex (cf. below). Here, activity in aSPL was found to co-vary with the index of absolute disparity. This appears somewhat puzzling considering that the “3D shapes contrast” in Durand's study should correspond to an increase of local gradients in the current study, rather than any change of absolute disparity.

Nonetheless, the peak of activation associated with the absolute disparity index in Exp 2 was in fact located more posteriorly in the SPL (pSPL). In this posterior region, our results are consistent with previous studies that used simple, but well-controlled visual stimuli. Specifically, Durand et al. [58] found that the posterior parietal cortex responded to the 3D-position condition, consistent with a representation of absolute disparity in the dorsal stream [12] (see also [11], which reported effects of absolute disparity in the medial bank of the posterior IPS). Our findings agree with the view that SPL includes several sub-regions for the processing of binocular disparity [63]. The search volume used in Exp 1 (aSPL) contained the sub-regions 7A and 7PC in cytoarchitectonic classification [64]. By contrast the main peak of activation associated with absolute disparity in Exp 2 was localized in cytoarchitectonic area 7P [64].

Together with these effects in the dorsal occipital cortex and the posterior parietal cortex, both the condition-based analysis of Exp 1 and the computational-based analyses of Exp 2 revealed disparity-related activations in the lateral occipital and posterior temporal cortices (see Figs. 2A and 3B). The block effect of 3D vs. 2D in Exp 1 showed activation of the lateral occipital cortex (LOC). Previous studies associated LOC with the representation of the 3D shape of objects (cf. [37]) and suggested that this region plays role in the computation of disparity gradients related to the surface geometry of 3D objects [29]–[31]. Here, we found that the gradient index affected activity in a more anterior region (pMTG). This was found in both Exp 2 and Exp 1, consistent with transient responses to time-varying disparity gradients in this region. Accordingly, we dissociated a sustained effect of 3D presentation in LOC vs. transient responses to dynamically changing local disparity gradients more anteriorly in the posterior temporal cortex (see also [61], which reported a posterior-anterior dissociation along the lateral occipito-temporal cortex related to the presence of a 3D surface vs. the subjective perception of that surface, using RDS moving stimuli).

Together with the gradient effect in pMTG, both Exp 2 and Exp 1 consistently showed that also activity in the left IFG co-varied with the disparity gradient index. The condition-based analysis of Exp 1 did not reveal any blocked effect of 3D vs. 2D in this region, indicating that the IFG responded only in a transient manner to time-varying changes local disparity signals. The activation was located in the left IFG including Brodmann areas BA44/45. This effect was somewhat unexpected, because studies using simple visual stimuli did not typically activate any such high level areas in the frontal cortex. Nonetheless, an fMRI study in non-human primates showed activation of area F5a during viewing of 3D shapes [65]. The activation of area F5a may correspond to the firing of the so-called “canonical neurons” that are thought to receive information about the stimulus location from the intra-parietal sulcus and about 3D shape from the inferior temporal cortex [66], [67]. The putative human homologue of monkey F5a (BA44) has been reported to show similar proprieties, with activation upon mere visual presentation of graspable objects (e.g. [68], [69]). In the current study, the gradient-related responses in the left IFG may correspond to the sensitivity of this region to objects' 3D shape properties. Because our protocol did not involve any motor output or motor planning, the effect in the left IFG may reflect object affordances beyond any explicit motor action [70].

The second set of findings of the current study relates to the use of complex auditory stimuli that were presented from multiple external sources (surround-sounds condition). A previous study using 45 external loudspeakers showed that naturalistic auditory stimuli presented from the speakers evoked larger and earlier brain responses comparing with the “artificial” auditory stimuli even using ITD and ILD [47]. Further, Callan and colleagues reported that sounds perceived to arise from external sources activated the posterior STG, including the PT, more than internalized sounds (i.e. sounds localized inside the head) [48]. These studies emphasize that realistic/naturalistic auditory stimuli that are localized in external space can lead to enhanced responses in the auditory cortex. Here, the surround stimulation with a multi-speakers system was also meant to increase the “spatial richness” of the auditory scene. The condition-based analysis associated the surround sounds with the activation of the auditory cortex and the planum temporale. However, it should be noted that the blocked stimulation with surround-sounds did not only entail an increase of sound “spatiality” but also an increase of the overall sound intensity. This could trivially explain the results of the condition-based analysis of Exp 1 (see also [6], [55]).

The computation-based approach, which we introduce here for the first time, allowed us to disentangle the contribution of sound spatiality and sound intensity. We sought to characterize the spatial richness of auditory scene by making use of a complexity index computed as the “difference” between the sounds played through each external speaker and the signal delivered over headphones. This index does not formally take into account spatial separation of the sources, but given that the sources were *de facto* spatially-separated we used variations of this index as a way to track the time-varying spatiality of the sounds in the surround condition (see also the discussion of [49], [50], and below). By definition the complexity index is unaffected by changes of intensity (cf. corresponding equation in the Methods section), nonetheless our computation-based analyses included 6 additional predictors seeking to fit variance associated with any intensity change during the surround stimulation.

The computation-based results of both Exp 2 and Exp 1 revealed that changes of auditory intensity affected activity in the STG/STS including PT, consistent with an overall effect of sound intensity in the surround condition (cf. condition-based results in Exp 1). Despite this, in Exp 2 we found that complexity index explained additional variance of the BOLD signal in PT, plus areas TE1.0 and TE1.2 in the Heschl's gyrus. By contrast, activity in area TE1.1 was unaffected by the sound complexity (see Fig. 4).

The finding of complexity/spatiality-related activity in PT is in agreement with the “dual route” model of auditory processing [71]–[73]. This model postulates a “where” pathway projecting from the primary auditory cortex to the posterior temporal and parietal cortices, and a different “what” pathway that instead projects to the anterior temporal cortex. The posterior pathway specializes in sound localization and sound motion detection, while the anterior pathway identifies auditory objects by processing spectro-temporal features [71]. In support of this model, many imaging studies of auditory space perception showed activation of the posterior “where” route (e.g. [49], [50], [74]–[79]).

Of particular relevance here is the work of Zatorre and colleagues, who made use of external sound sources during PET scanning [49], [50]. In a first set of studies [49], the authors showed increased activation in PT when naturalistic stimuli were presented from a spatially distributed range of locations. The protocol disentangled the “spatiality” and the “complexity” of the auditory scene by varying the number of locations but keeping the number of stimuli constant (n = 12). In a subsequent study, the same authors varied the number of complex sounds (n between 1 and 45) that were now presented from a single external location [50]. This experiment did not reveal any co-variation between activity in PT and the number of sounds, showing instead a negative relationship between the number of stimuli and activity in anterior temporal regions. Taken together these two studies indicate that activity in PT reflects the spatial distribution of sounds (i.e. “spatiality”), rather than the mere “amount” of auditory information, substantiating the interpretation of our “complexity index” as a measure of the time-varying spatial richness during surround stimulation. Accordingly, we reckon that the computation-based analyses of the surround-sound stimuli allowed us to track processing of auditory spatial signals in complex naturalistic environments, also accounting for any effect related to changing sound intensity.

Nonetheless, we acknowledge that the sounds delivered via the multi-speakers system in the MR scanner are likely to generate changes along several acoustic dimensions, other than just complexity/spatiality and intensity. First, the piezo-speakers have a relatively poor response at low frequency (<450 Hz), which is a limitation of the setup used here. Second, we used a neoprene foam sheet to attenuate reverberations within the MR bore. The sheet was placed on the head-coil and reduced the echoes from the front side. However, the noise from the top/back side could not be reduced, because this would have obstructed the viewing of the screen for the 3D projection. Thus, sounds from the multi-speaker system reverberated within the MR bore, reducing the overall sound quality and possibly acting as a low-pass filter. Future developments of the multi-speakers system will include using additional material for acoustic isolation (e.g. by adding foam sheet on the bore of scanner).

While these factors/limitations are likely to have contributed to the pattern of activation observed when comparing “surround vs. mono” conditions in Exp 1, it is less clear whether/how these also affected the results of the computation-based analyses of Exp 2. These did not compare conditions “with vs. without” the multi-speakers system, but rather used variance over periods that always included surround-stimuli and – possibly – the associated echoes, reverberations, low-pass filtering, etc. Further, the complexity index reflected the relationship (i.e. angular difference, see methods section) between the signal of the sound-sources (each speaker vs. headphone), rather than the specific sound characteristics (e.g. spectral density) of a single source/speaker. Finally, the “complexity” index is formally independent from sound intensity, thus complexity can be high when the sound intensity and, presumably, echoes and reverberation in the MR bore were low. Nonetheless, we cannot exclude that the surround stimulation generated changes over time of some other acoustic factor that co-varied with “complexity” index, thus contributing to the effects in STG and STS that we found in Exp 2 (see also [40] and [80]).

In the last decade, several computation-based approaches have been successfully used to investigate brain activity during presentation of naturalistic stimuli (e.g. [3], [4], [55]). Computation-based analysis can identify brain areas where the hemodynamic response co-varies with stimulus indexes derived from the computational models. Here, we applied this approach to investigate the processing of binocular disparity and multi-sources sounds in 3D-surround cinematography. We found that activity in area V3A showed both sustained responses to stereoscopic 3D and dynamic changes co-varying with absolute disparity. The SPL and LOC also responded to blocked 3D stimulation, with the SPL showing an effect of absolute disparity primarily in its posterior division (pSPL). By contrast, the pMTG and the left IFG did not show any blocked effect of 3D vs. 2D presentation, but showed time-varying signal changes correlating with disparity gradients. Computation-based analyses of the multi-sources surround sounds associated the processing of scene spatiality with the activity in PT, even after accounting for any change of sound intensity.

Although naturalistic stimuli cannot replace well-controlled experimental protocols [7], our results highlight that these can not only confirm the result obtained with standard protocols but can also help identifying novel aspects of stimulus processing, which may then guide the design of new experiments with standardized stimuli. Here we demonstrate that the combination of functional neuroimaging and computation-based analyses of naturalistic stimuli can reveal brain activity associated with the processing of three-dimensional, surround-sound cinematography.

## References

[1] Sanchez-Vives MV, Slater M (2005) From presence to consciousness through virtual reality. Nat Rev Neurosci 6: 332–339. 15803164 10.1038/nrn1651

[2] Hasson U, Furman O, Clark D, Dudai Y, Davachi L (2008) Enhanced intersubject correlations during movie viewing correlate with successful episodic encoding. Neuron 57: 452–462. 18255037 10.1016/j.neuron.2007.12.009 PMC2789242

[3] Bartels A, Zeki S (2004) Functional brain mapping during free viewing of natural scenes. Hum Brain Mapp 21: 75–85. 14755595 10.1002/hbm.10153 PMC6872023

[4] Bartels A, Zeki S, Logothetis NK (2008) Natural vision reveals regional specialization to local motion and to contrast-invariant, global flow in the human brain. Cereb Cortex 18: 705–717. 17615246 10.1093/cercor/bhm107

[5] Fiser J, Chiu C, Weliky M (2004) Small modulation of ongoing cortical dynamics by sensory input during natural vision. Nature 431: 573–578. 15457262 10.1038/nature02907

[6] Kayser C, Körding KP, König P (2004) Processing of complex stimuli and natural scenes in the visual cortex. Curr Opin Neurobiol 14: 468–473. 15302353 10.1016/j.conb.2004.06.002

[7] Rust NC, Movshon JA (2005) In praise of artifice. Nat Neurosci 8: 1647–1650. 16306892 10.1038/nn1606

[8] Qian N (1997) Binocular disparity and the perception of depth. Neuron 18: 359–368. 9115731 10.1016/s0896-6273(00)81238-6

[9] Anzai A, DeAngelis GC (2010) Neural computations underlying depth perception. Curr Opin Neurobiol 20: 367–375. 20451369 10.1016/j.conb.2010.04.006 PMC2883007

[10] Read J (2005) Early computational processing in binocular vision and depth perception. Prog Biophys Mol Biol 87: 77–108. 15471592 10.1016/j.pbiomolbio.2004.06.005 PMC1414095

[11] Tsao DY, Vanduffel W, Sasaki Y, Fize D, Knutsen TA, et al (2003) Stereopsis activates V3A and caudal intraparietal areas in macaques and humans. Neuron 39: 555–568. 12895427 10.1016/s0896-6273(03)00459-8

[12] Neri P, Bridge H, Heeger DJ (2004) Stereoscopic processing of absolute and relative disparity in human visual cortex. J Neurophysiol 92: 1880–1891. 15331652 10.1152/jn.01042.2003

[13] Backus BT, Fleet DJ, Parker AJ, Heeger DJ (2001) Human cortical activity correlates with stereoscopic depth perception. J Neurophysiol 86: 2054–2068. 11600661 10.1152/jn.2001.86.4.2054

[14] Anzai A, Chowdhury SA, DeAngelis GC (2011) Coding of stereoscopic depth information in visual areas V3 and V3A. J Neurosci 31: 10270–10282. 21753004 10.1523/JNEUROSCI.5956-10.2011 PMC3143190

[15] Parker AJ (2007) Binocular depth perception and the cerebral cortex. Nat Rev Neurosci 8: 379–391. 17453018 10.1038/nrn2131

[16] Poggio GF, Gonzalez F, Krause F (1988) Stereoscopic mechanisms in monkey visual cortex: binocular correlation and disparity selectivity. J Neurosci 8: 4531–4550. 3199191 10.1523/JNEUROSCI.08-12-04531.1988 PMC6569547

[17] Preston TJ, Kourtzi Z, Welchman AE (2009) Adaptive estimation of three-dimensional structure in the human brain. J Neurosci 29: 1688–1698. 19211876 10.1523/JNEUROSCI.5021-08.2009 PMC6666271

[18] Sakata H, Tsutsui K, Taira M (2005) Toward an understanding of the neural processing for 3D shape perception. Neuropsychologia 43: 151–161. 15707901 10.1016/j.neuropsychologia.2004.11.003

[19] Taira M, Tsutsui KI, Jiang M, Yara K, Sakata H (2000) Parietal neurons represent surface orientation from the gradient of binocular disparity. J Neurophysiol 83: 3140–3146. 10805708 10.1152/jn.2000.83.5.3140

[20] Tsutsui K, Jiang M, Yara K, Sakata H, Taira M (2001) Integration of perspective and disparity cues in surface-orientation-selective neurons of area CIP. J Neurophysiol 86: 2856–2867. 11731542 10.1152/jn.2001.86.6.2856

[21] Tsutsui K, Sakata H, Naganuma T, Taira M (2002) Neural correlates for perception of 3D surface orientation from texture gradient. Science 298: 409–412. 12376700 10.1126/science.1074128

[22] Janssen P, Vogels R, Liu Y, Orban GA (2001) Macaque inferior temporal neurons are selective for three-dimensional boundaries and surfaces. J Neurosci 21: 9419–9429. 11717375 10.1523/JNEUROSCI.21-23-09419.2001 PMC6763913

[23] Janssen P, Vogels R, Orban GA (1999) Macaque inferior temporal neurons are selective for disparity-defined three-dimensional shapes. Proc Natl Acad Sci U S A 96: 8217–8222. 10393975 10.1073/pnas.96.14.8217 PMC22215

[24] Janssen P, Vogels R, Orban GA (2000) Three-dimensional shape coding in inferior temporal cortex. Neuron 27: 385–397. 10985357 10.1016/s0896-6273(00)00045-3

[25] Malach R, Reppas JB, Benson RR, Kwong KK, Jiang H, et al (1995) Object-related activity revealed by functional magnetic resonance imaging in human occipital cortex. Proc Natl Acad Sci U S A 92: 8135–8139. 7667258 10.1073/pnas.92.18.8135 PMC41110

[26] Denys K, Vanduffel W, Fize D, Nelissen K, Peuskens H, et al (2004) The processing of visual shape in the cerebral cortex of human and nonhuman primates: a functional magnetic resonance imaging study. J Neurosci 24: 2551–2565. 15014131 10.1523/JNEUROSCI.3569-03.2004 PMC6729498

[27] Grill-Spector K (2003) The neural basis of object perception. Curr Opin Neurobiol 13: 159–166. 12744968 10.1016/s0959-4388(03)00040-0

[28] Kourtzi Z, Kanwisher N (2001) Representation of perceived object shape by the human lateral occipital complex. Science 293: 1506–1509. 11520991 10.1126/science.1061133

[29] Chandrasekaran C, Canon V, Dahmen JC, Kourtzi Z, Welchman AE (2007) Neural correlates of disparity-defined shape discrimination in the human brain. J Neurophysiol 97: 1553–1565. 17151220 10.1152/jn.01074.2006

[30] Gilaie-Dotan S, Ullman S, Kushnir T, Malach R (2002) Shape-selective stereo processing in human object-related visual areas. Hum Brain Mapp 15: 67–79. 11835599 10.1002/hbm.10008 PMC6872042

[31] Cumming B (2002) Stereopsis: where depth is seen. Curr Biol 12: R93–95. 11839288 10.1016/s0960-9822(02)00669-3

[32] Ban H, Preston TJ, Meeson A, Welchman AE (2012) The integration of motion and disparity cues to depth in dorsal visual cortex. Nat Neurosci 15: 636–643. 22327475 10.1038/nn.3046 PMC3378632

[33] Preston TJ, Li S, Kourtzi Z, Welchman AE (2008) Multivoxel pattern selectivity for perceptually relevant binocular disparities in the human brain. J Neurosci 28: 11315–11327. 18971473 10.1523/JNEUROSCI.2728-08.2008 PMC6671500

[34] Krug K, Cumming BG, Parker AJ (2004) Comparing perceptual signals of single V5/MT neurons in two binocular depth tasks. J Neurophysiol 92: 1586–1596. 15102899 10.1152/jn.00851.2003

[35] Uka T, DeAngelis GC (2006) Linking neural representation to function in stereoscopic depth perception: roles of the middle temporal area in coarse versus fine disparity discrimination. J Neurosci 26: 6791–6802. 16793886 10.1523/JNEUROSCI.5435-05.2006 PMC1994558

[36] Georgieva SS, Todd JT, Peeters R, Orban GA (2008) The extraction of 3D shape from texture and shading in the human brain. Cereb Cortex 18: 2416–2438. 18281304 10.1093/cercor/bhn002 PMC2536698

[37] Welchman AE, Deubelius A, Conrad V, Bülthoff HH, Kourtzi Z (2005) 3D shape perception from combined depth cues in human visual cortex. Nat Neurosci 8: 820–827. 15864303 10.1038/nn1461

[38] Alink A, Euler F, Kriegeskorte N, Singer W, Kohler A (2012) Auditory motion direction encoding in auditory cortex and high-level visual cortex. Hum Brain Mapp 33: 969–978. 21692141 10.1002/hbm.21263 PMC6870293

[39] Deouell LY, Heller AS, Malach R, D'Esposito M, Knight RT (2007) Cerebral responses to change in spatial location of unattended sounds. Neuron 55: 985–996. 17880900 10.1016/j.neuron.2007.08.019

[40] Kopco N, Huang S, Belliveau JW, Raij T, Tengshe C, et al (2012) Neuronal representations of distance in human auditory cortex. Proc Natl Acad Sci U S A 109: 11019–11024. 22699495 10.1073/pnas.1119496109 PMC3390865

[41] Lewald J, Riederer KA, Lentz T, Meister IG (2008) Processing of sound location in human cortex. Eur J Neurosci 27: 1261–1270. 18364040 10.1111/j.1460-9568.2008.06094.x

[42] Warren JD, Uppenkamp S, Patterson RD, Griffiths TD (2003) Separating pitch chroma and pitch height in the human brain. Proc Natl Acad Sci U S A 100: 10038–10042. 12909719 10.1073/pnas.1730682100 PMC187755

[43] Altmann CF, Henning M, Döring MK, Kaiser J (2008) Effects of feature-selective attention on auditory pattern and location processing. Neuroimage 41: 69–79. 18378168 10.1016/j.neuroimage.2008.02.013

[44] Zündorf IC, Lewald J, Karnath HO (2013) Neural correlates of sound localization in complex acoustic environments. PLoS One 8: e64259. 23691185 10.1371/journal.pone.0064259 PMC3653868

[45] Geschwind N, Levitsky W (1968) Human brain: left-right asymmetries in temporal speech region. Science 161: 186–187. 5657070 10.1126/science.161.3837.186

[46] Rauschecker JP, Tian B, Hauser M (1995) Processing of complex sounds in the macaque nonprimary auditory cortex. Science 268: 111–114. 7701330 10.1126/science.7701330

[47] Getzmann S, Lewald J (2010) Effects of natural versus artificial spatial cues on electrophysiological correlates of auditory motion. Hear Res 259: 44–54. 19800957 10.1016/j.heares.2009.09.021

[48] Callan A, Callan DE, Ando H (2012) Neural correlates of sound externalization. Neuroimage 66C: 22–27. 10.1016/j.neuroimage.2012.10.057 23108271

[49] Zatorre RJ, Bouffard M, Ahad P, Belin P (2002) Where is ‘where’ in the human auditory cortex? Nat Neurosci 5: 905–909. 12195426 10.1038/nn904

[50] Zatorre RJ, Bouffard M, Belin P (2004) Sensitivity to auditory object features in human temporal neocortex. J Neurosci 24: 3637–3642. 15071112 10.1523/JNEUROSCI.5458-03.2004 PMC6729744

[51] Brainard DH (1997) The Psychophysics Toolbox. Spat Vis 10: 433–436. 9176952

[52] Liu C, Yuen J (2011) SIFT Flow: Dense correspondence across scenes and its applications. IEEE T Pattern Anal 33: 978–994. 10.1109/TPAMI.2010.147 20714019

[53] Lowe DG (1999) Object recognition from local scale-invariant features. Proceedings of the International Conference on Computer Vision, pp. 1150–1157.

[54] Greenspan H, Belongie S, Goodman R, Perona P, Rakshit S, et al. (1994) Overcomplete steerable pyramid filters and rotation invariance, The IEEE Conference on Computer Vision and Pattern Recognition, pp. 222–228.

[55] Bordier C, Puja F, Macaluso E (2013) Sensory processing during viewing of cinematographic material: Computational modeling and functional neuroimaging. Neuroimage 67: 213–226. 23202431 10.1016/j.neuroimage.2012.11.031 PMC3838951

[56] Singhal A (2001) Modern information retrieval: A brief overview. Bulletin of the IEEE Computer Society Technical Committee on Data Engineering 24: 35–43.

[57] Shamma S (2001) On the role of space and time in auditory processing. Trends Cogn Sci 5: 340–348. 11477003 10.1016/s1364-6613(00)01704-6

[58] Durand JB, Peeters R, Norman JF, Todd JT, Orban GA (2009) Parietal regions processing visual 3D shape extracted from disparity. Neuroimage 46: 1114–1126. 19303937 10.1016/j.neuroimage.2009.03.023

[59] Poldrack RA (2007) Region of interest analysis for fMRI. Soc Cogn Affect Neurosci 2: 67–70. 18985121 10.1093/scan/nsm006 PMC2555436

[60] Swisher JD, Halko MA, Merabet LB, McMains SA, Somers DC (2007) Visual topography of human intraparietal sulcus. J Neurosci 27: 5326–5337. 17507555 10.1523/JNEUROSCI.0991-07.2007 PMC6672354

[61] Beer AL, Watanabe T, Ni R, Sasaki Y, Andersen GJ (2009) 3D surface perception from motion involves a temporal-parietal network. Eur J Neurosci 30: 703–713. 19674088 10.1111/j.1460-9568.2009.06857.x PMC2902171

[62] Minini L, Parker AJ, Bridge H (2010) Neural modulation by binocular disparity greatest in human dorsal visual stream. J Neurophysiol 104: 169–178. 20445027 10.1152/jn.00790.2009 PMC2904223

[63] Abdollahi RO, Jastorff J, Orban GA Common and segregated processing of observed actions in human SPL. Cereb Cortex In press. 10.1093/cercor/bhs264 22918981

[64] Scheperjans F, Hermann K, Eickhoff SB, Amunts K, Schleicher A, et al (2008) Observer-independent cytoarchitectonic mapping of the human superior parietal cortex. Cereb Cortex 18: 846–867. 17644831 10.1093/cercor/bhm116

[65] Joly O, Vanduffel W, Orban GA (2009) The monkey ventral premotor cortex processes 3D shape from disparity. Neuroimage 47: 262–272. 19376235 10.1016/j.neuroimage.2009.04.043

[66] Gallese V, Fadiga L, Fogassi L, Rizzolatti G (1996) Action recognition in the premotor cortex. Brain 119 (Pt 2) 593–609. 8800951 10.1093/brain/119.2.593

[67] Rizzolatti G, Fadiga L, Gallese V, Fogassi L (1996) Premotor cortex and the recognition of motor actions. Brain Res Cogn Brain Res 3: 131–141. 8713554 10.1016/0926-6410(95)00038-0

[68] Grèzes J, Armony JL, Rowe J, Passingham RE (2003) Activations related to “mirror” and “canonical” neurones in the human brain: an fMRI study. Neuroimage 18: 928–937. 12725768 10.1016/s1053-8119(03)00042-9

[69] Press C, Weiskopf N, Kilner JM (2012) Dissociable roles of human inferior frontal gyrus during action execution and observation. Neuroimage 60: 1671–1677. 22321646 10.1016/j.neuroimage.2012.01.118 PMC3399774

[70] Costantini M, Ambrosini E, Tieri G, Sinigaglia C, Committeri G (2010) Where does an object trigger an action? An investigation about affordances in space. Exp Brain Res 207: 95–103. 20931177 10.1007/s00221-010-2435-8

[71] Rauschecker JP, Tian B (2000) Mechanisms and streams for processing of “what” and “where” in auditory cortex. Proc Natl Acad Sci U S A 97: 11800–11806. 11050212 10.1073/pnas.97.22.11800 PMC34352

[72] Romanski LM, Tian B, Fritz J, Mishkin M, Goldman-Rakic PS, et al (1999) Dual streams of auditory afferents target multiple domains in the primate prefrontal cortex. Nat Neurosci 2: 1131–1136. 10570492 10.1038/16056 PMC2778291

[73] Arnott SR, Binns MA, Grady CL, Alain C (2004) Assessing the auditory dual-pathway model in humans. Neuroimage 22: 401–408. 15110033 10.1016/j.neuroimage.2004.01.014

[74] Maeder PP, Meuli RA, Adriani M, Bellmann A, Fornari E, et al (2001) Distinct pathways involved in sound recognition and localization: a human fMRI study. Neuroimage 14: 802–816. 11554799 10.1006/nimg.2001.0888

[75] Warren JD, Zielinski BA, Green GG, Rauschecker JP, Griffiths TD (2002) Perception of sound-source motion by the human brain. Neuron 34: 139–148. 11931748 10.1016/s0896-6273(02)00637-2

[76] Krumbholz K, Schönwiesner M, Rübsamen R, Zilles K, Fink GR, et al (2005) Hierarchical processing of sound location and motion in the human brainstem and planum temporale. Eur J Neurosci 21: 230–238. 15654860 10.1111/j.1460-9568.2004.03836.x

[77] Zimmer U, Macaluso E (2005) High binaural coherence determines successful sound localization and increased activity in posterior auditory areas. Neuron 47: 893–905. 16157283 10.1016/j.neuron.2005.07.019

[78] Alain C, Arnott SR, Hevenor S, Graham S, Grady CL (2001) “What” and “where” in the human auditory system. Proc Natl Acad Sci U S A 98: 12301–12306. 11572938 10.1073/pnas.211209098 PMC59809

[79] Warren JD, Griffiths TD (2003) Distinct mechanisms for processing spatial sequences and pitch sequences in the human auditory brain. J Neurosci 23: 5799–5804. 12843284 10.1523/JNEUROSCI.23-13-05799.2003 PMC6741275

[80] Lahnakoski JM, Salmi J, Jääskeläinen IP, Lampinen J, Glerean E, et al (2012) Stimulus-related independent component and voxel-wise analysis of human brain activity during free viewing of a feature film. PLoS One 7: e35215. 22496909 10.1371/journal.pone.0035215 PMC3320648

[81] Brett M, Anton J-L, Valabregue R, Poline J-B (2002) Region of interest analysis using an SPM toolbox, Organization for Human Brain Mapping annual meeting, Sendai, Japan.

[82] Eickhoff SB, Stephan KE, Mohlberg H, Grefkes C, Fink GR, et al (2005) A new SPM toolbox for combining probabilistic cytoarchitectonic maps and functional imaging data. Neuroimage 25: 1325–1335. 15850749 10.1016/j.neuroimage.2004.12.034

